# Mobile-bearing insert used with total knee arthroplasty does not rotate on the tibial tray during a squatting activity: a cross-sectional study

**DOI:** 10.1186/s13018-020-1570-6

**Published:** 2020-03-20

**Authors:** Kenji Hoshi, Goro Watanabe, Yasuo Kurose, Ryuji Tanaka, Jiro Fujii, Kazuyoshi Gamada

**Affiliations:** 1 Graduate School of Medical Technology and Health Welfare Sciences, Hiroshima International University, 555-36 Kurose-Gakuendai, Bldg3, Rm3807, Higashi-Hiroshima, Hiroshima 739-2695 Japan; 2 Hiroshima Prefectural Rehabilitation Center, 295-3 Taguchi, Saijo-cho, Higashi-Hiroshima, Hiroshima 739-0036 Japan

**Keywords:** Total knee arthroplasty, Kinematics, Knee joint, Rotation

## Abstract

**Background:**

Total knee arthroplasty (TKA) is commonly performed around the world. Implant designs include fixed-bearing and mobile-bearing. Mobile-bearing design was developed as a rotating platform that allows axial rotation of the insert around the longitudinal axis. This phenomenon may limit full exploitation of the characteristics of the mobile-bearing insert, which may cause wearing and reduce longevity. However, there is limited knowledge on rotational behavior of the polyethylene mobile-bearing insert under weight-bearing conditions. We aimed at determining the rotational motion of each component at full extension and flexed positions during a squatting activity after TKA.

**Methods:**

This study was a cross-sectional study (level 4) involving patients with severe knee osteoarthritis scheduled to receive TKA. We examined 13 knees of 11 patients after mobile-bearing TKA (NexGen LPS-Flex, Zimmer Inc.) at 10 weeks and 1 year postoperatively. Four identical metallic beads were embedded into the insert. Wide-base squatting was chosen for analyses. Three-dimensional in vivo poses of the prostheses were created using a 3D-to-2D registration technique. During flexion, rotation of the femoral component relative to the insert (FEM/INS) and tibial component (FEM/TIB) as well as insert rotation relative to the tibial component (INS/TIB) were computed. Repeated measure 2-way ANOVA and post hoc test was used.

**Results:**

In the fully extended position, FEM/INS was significantly smaller than INS/TIB both at 10 weeks (− 0.3° vs. 6.3°, *p* = .013) and 1 year (− 0.8° vs. 4.9°, *p* = .011), respectively. During the squatting activity, rotation motions of FEM/TIB, FEM/INS, INS/TIB were 5.7°, 5.9°, and 1.8° at 10 weeks and 6.3°, 5.5°, and 1.6° at 1 year, respectively. Rotation motion of FEM/INS was significantly greater than that of INS/TIB at both 10 weeks (*p* < .001) and 1 year (*p* < .001).

**Conclusions:**

The mobile-bearing insert enhances the compatibility of FEM/INS in extension; the amount of INS/TIB rotation is significantly smaller than that of FEM/INS during a squatting activity. This information will inform surgeons to take caution to perform TKA with a fixed insert in which 6.3° of rotational offset would be added to the rotational alignment at FEM/INS at full extension.

**Trial registration:**

UMIN-CTR, UMIN000024196. Retrospectively registered on 9 September 2016.

## Background

The design of the insert of the artificial joint can be roughly divided into two types, fixed-bearing and mobile-bearing. Mobile-bearing design was developed as a rotating platform that allows axial rotation of the insert around the longitudinal axis. It was theorized that mobile-bearing total knee arthroplasty (TKA) should allow self-alignment of the polyethylene insert with the femoral component in order to lessen polyethylene surface stresses, minimize post-cam impingement, and possibly increase polyethylene longevity [1]. However, many studies failed to prove that mobile-bearing insert contributed to reduce insert wear or risk of loosening, as well as to improve clinical outcomes such as range of motion, functional scores, and pain relief [2–10]. Moreover, none of the studies utilizing an accurate kinematic analysis system have proven that femoral-tibial component kinematics differs between mobile-bearing and fixed-bearing inserts [11–13]. However, precise movement of the mobile-bearing insert has not been clarified and the usefulness of the mobile-bearing insert in vivo remains uncertain.

Several studies have used embedded tantalum beads to analyze the behavior of the mobile-bearing insert by X-ray fluoroscopy during in vivo knee motion [14–17]. These studies demonstrated that axial rotation of the femoral component with respect to the mobile insert was small. Moreover, the rotation offset, which refers to the difference in the rotational positioning between two components, during flexion was limited [15]. However, Dennis et al. [18] demonstrated that the behavior of insert rotation after mobile-bearing TKA is highly variable and depends on the implant design. In the NexGen LPS mobile-bearing knee prosthesis (Zimmer Inc. Warsaw, IN, USA), there is a limited degree of conformity of the femoral component on the insert surface, allowing the femoral component to slide with respect to the insert (± 12° of rotation) [19]. Garling et al. [19] showed that, in patients with rheumatoid arthritis, the conformity of the NexGen LPS mobile bearing knee prosthesis was low enough that the femoral component was allowed to translate with respect to the insert without forcing the insert to rotate. Consequently, we can speculate that, if the conformity of the mobile-bearing insert is low, the femoral component can rotate and slide on the insert more than insert rotation relative to the tibial tray. Therefore, the mobile-bearing insert with low conformity between the femoral component and insert may limit its ability to rotate on the tibial tray, which may reduce longevity. Due to the lack of knowledge on the behavior of the mobile-bearing in patients with knee osteoarthritis (KOA), there has not been any consensus on the behavior of the mobile-bearing insert in patients with KOA.

The objective of this study was to clarify the tibiofemoral kinematics and rotation of the polyethylene mobile-bearing insert relative to the femoral and tibial components under weight-bearing conditions in patients implanted with mobile-bearing TKA. The hypotheses of this study were as follows: (1) the mobile-bearing insert can rotate on the tibial tray to reduce the offset at FEM/INS at knee extension position and (2) intra-prosthetic axial rotation between the tibial tray and mobile-bearing insert is greater than that between the femoral component and mobile-bearing insert.

## Methods

### Participants

This study was a cross-sectional study (Level 4) involving patients with severe knee osteoarthritis scheduled to receive TKA at a single regional hospital. The study protocol was approved by the institutional review board of Hiroshima International University (12-025), and informed consent was obtained from all subjects included in the study prior to the initiation of this study. All subjects who met specific selection criteria were enrolled. Inclusion criteria were (a) Japanese males and females and (b) between 50 and 85 years old, primary medial KOA with radiographic severity of grade III or IV in the Kellgren-Lawrence system, with or without lateral or patellofemoral degradation. Exclusion criteria were (a) lateral compartment knee osteoarthritis; (b) history of knee surgery; (c) any other disease involving the knee joint; and (d) history of rheumatoid arthritis, cerebral disorders, neuropathy, or gout.

### Surgical technique and procedure to embed tantalum beads

All osteoarthritic knees underwent TKA with posterior stabilizing (PS) components and mobile-bearing insert (NexGen LPS-Flex, Mobile Surface, Zimmer Inc., Warsaw, IN, USA). This prosthesis was designed to safely allow 155° flexion and has a mobile bearing that allows up to 25° internal or external rotation on an anteriorly placed rotation center. A senior surgeon (YK) performed arthroplasty using his own technique, which is summarized by a paramedian approach for minimal exposure, achieving 6° valgus for the femoral cut and 90° to the tibial shaft, modified ligament dependent cut for femoral rotational alignment.

**Fig. 1:**
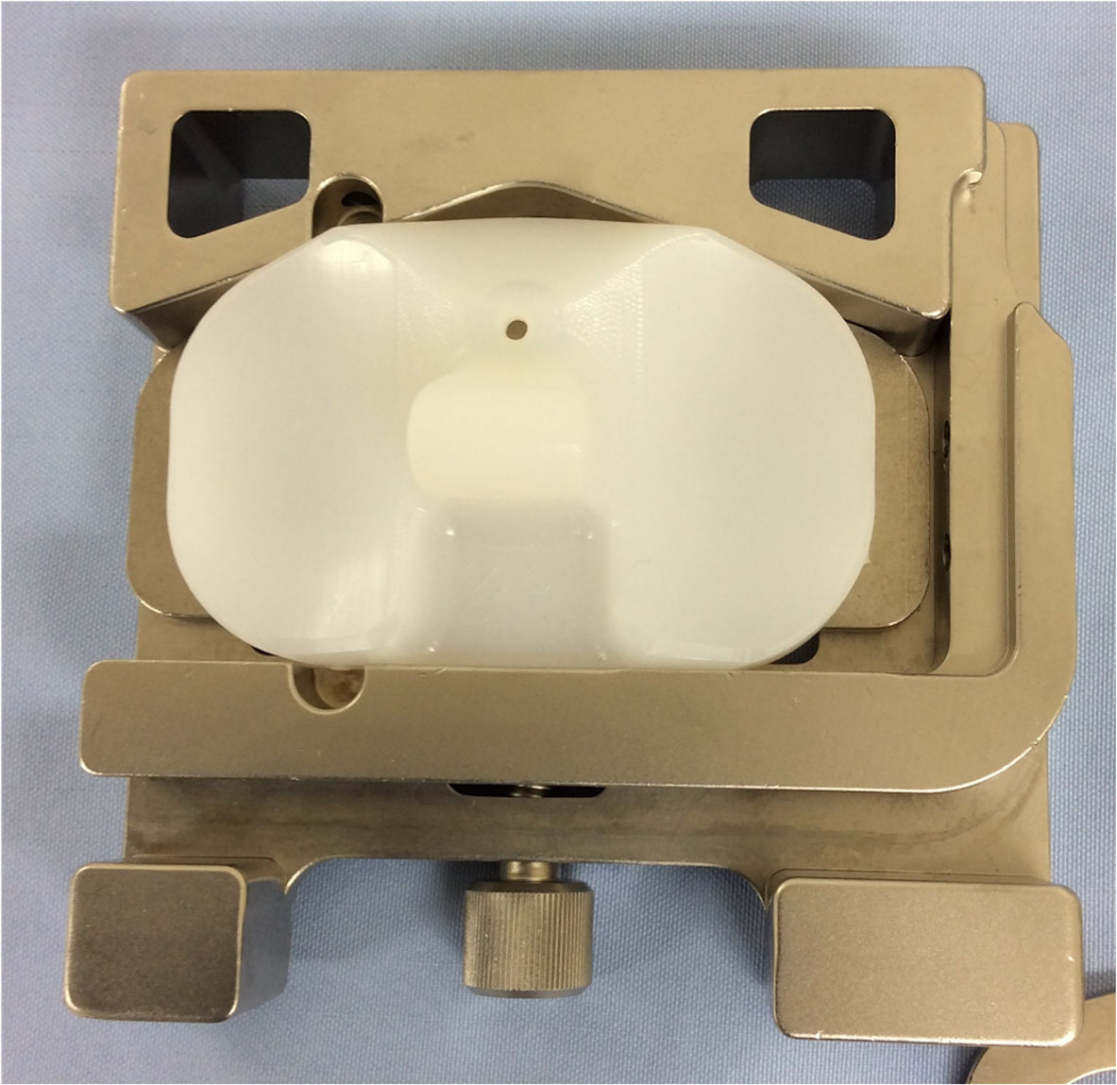
A custom-made insert holder has four holes, through which a beed injecter was inserted to shoot beeds at reproducible locations

Because the polyethylene insert is transparent during X-ray fluoroscopy, three-dimensional pose estimation of the mobile-bearing insert was achieved using tantalum beads embedded in the insert. Using a custom-made bead injector, four identical metallic tantalum beads (diameter 0.8 mm) were inserted into predefined non-weight-bearing areas of the polyethylene insert during surgery (Fig. 1). The four holes in the external surface of the template device were the same size as the head of the bead injector, and the tantalum beads were precisely inserted at a constant height and depth from the lateral surface of the insert. Since the actual positions of the embedded tantalum beads must be confirmed to minimize errors in beads positioning, we performed CT imaging on all subjects on a different day from surgery to determine the exact beads positions, and a subject-specific computer-added design (CAD) model of the four beads (bead model) was created in each case. All subjects received same postoperative treatment and rehabilitation program.

### Outcomes

We measured active and passive knee flexion angles, Western Ontario and McMaster Universities Osteoarthritis Index (WOMAC) [20], and articular component kinematics during a squatting activity at 10 weeks and 1 year postoperatively. We used pain, stiffness, physical function scores and total score of 100 mm visual analogue scale WOMAC. A single examiner obtained all clinical and kinematic measurements.

### Clinical outcomes

Weight bearing and passive knee range of motion (ROM) were measured using sagittal images from a movie taken with a commercial video camera and analyzed using the ImageJ software (ImageJ 1.48v, National Institutes of Health, Bethesda, MD). The video camera was positioned 3 m away from the subject to bilateral sides and adjusted the camera so that the center of the knee joint was at the center of the viewing screen. For the weight bearing ROM, subjects stood sideway to the camera and were instructed to squat down from full extension to maximal flexion with maximal effort. They were allowed to hold handrail for safety. For passive knee flexion measurement, subjects were instructed to lie supine on a bed, and a senior physical therapist bent their knee to maximum flexion.

Symptoms and physical function were measured using the WOMAC index at 10 weeks and again 1 year postoperatively. Subjects responded to all sections including subscales of pain, stiffness, and physical function during daily living. All measurements were performed by an 11th year physical therapist with research experience of 4 years as a graduate student.

### Model registration and data processing

**Fig. 2:**
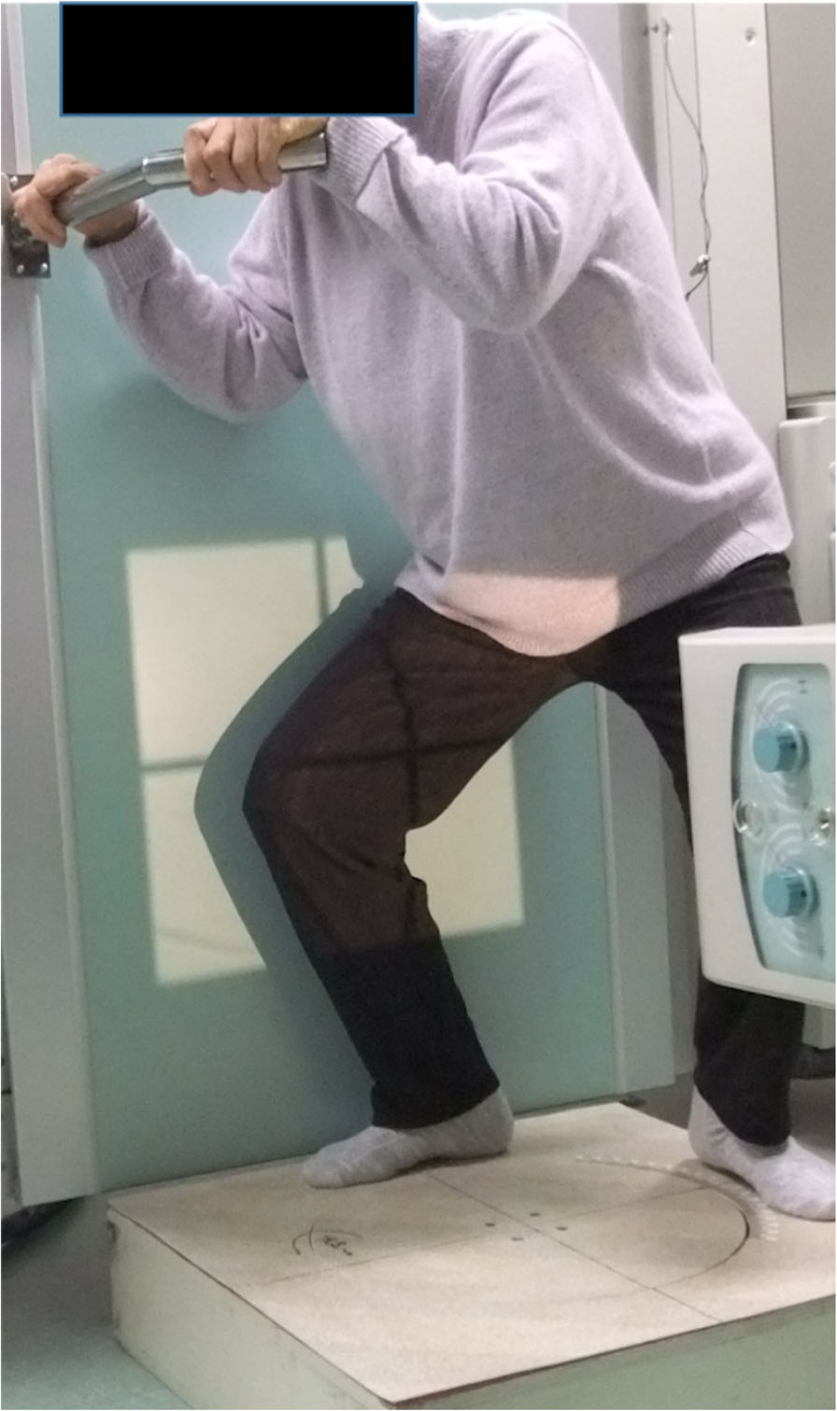
Patients were instructed to stand with his or her hips externally rotated with the feet angled at 90° to avoid overlapping of the contralateral knee. They were allowed to hold an handrail for safety

We chose wide-based squat for articular kinematic analyses. Each subject was asked to perform the wide-base squat during fluoroscopic surveillance using a 17-in. flat pannel (15 Hz, SONIALVISION Safire 17, Shimazu Corp., Japan). To avoid the influence of shoes and insole, all images were obtained while the subject was barefoot. Subjects were instructed to squat as slowly as possible with the hips externally rotated so that the feet were angled at 90^o^ [21] (Fig. 2). Subjects were instructed to bend taking more than 5 s and extent more than 5 s. In this position, overlapping of the contralateral knee on the fluoroscopic view could be avoided. As noted above, subjects were allowed to hold onto handrail for safety. However, they were instructed not to allow forward movement of the knee beyond the toes while squatting in order to avoid knee movement outside of the fluoroscopic view. Subjects practiced the activity until they felt comfortable, and the motion became smooth before recording began. Successful knee motions were recorded as serial digital X-ray images.

**Fig. 3:**
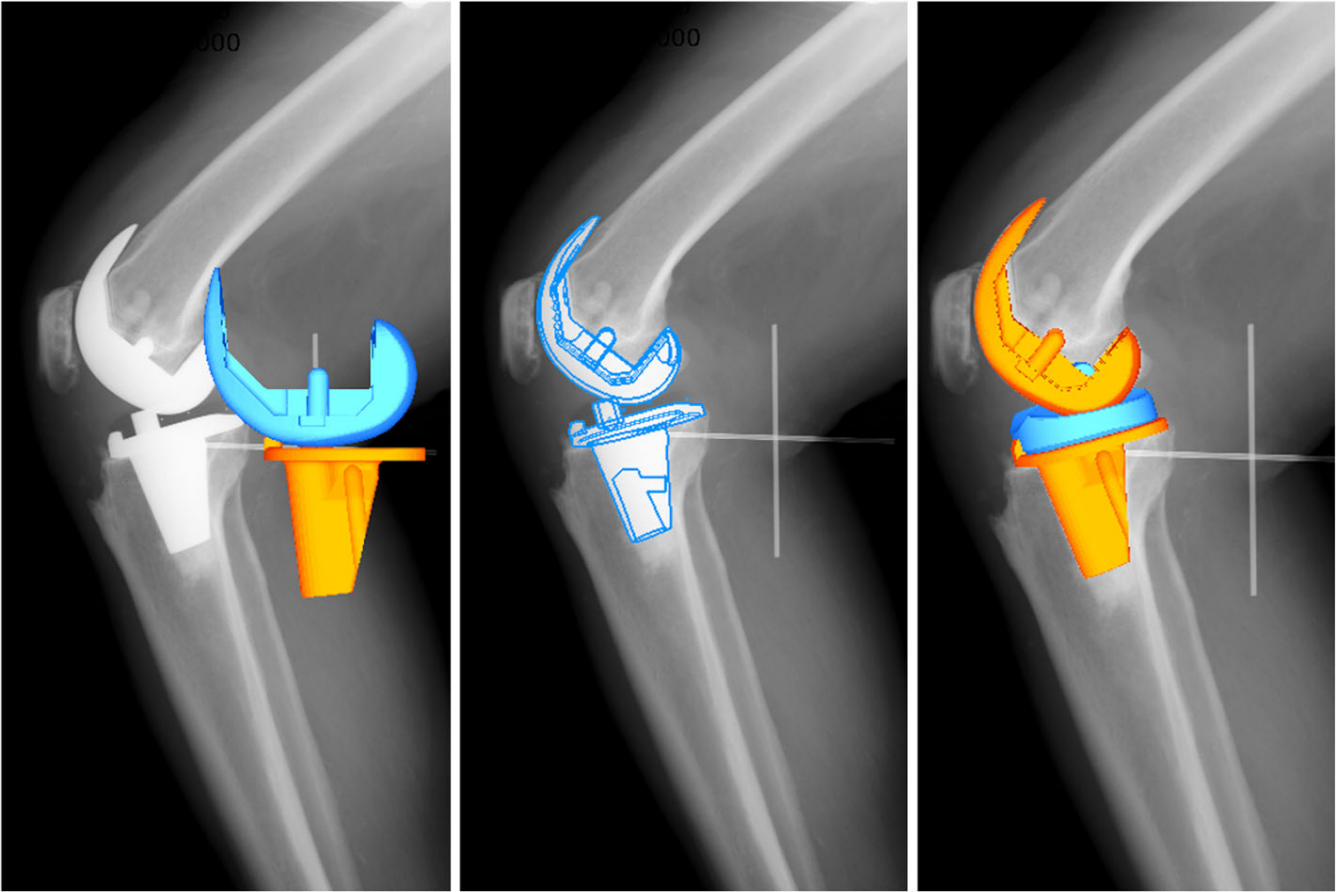
3-dimensional CAD models were laid over the calibrated fluoroscopic images to determine the best-fitted position

All analyses were performed by a single experienced examiner. Three-dimensional in vivo images of the prostheses were obtained using a 3D-to-2D registration technique [22]. Calibrated fluoroscopic images and CAD models of the femoral and tibial components, as well as the bead model composed of the four tantalum beads implanted in the polyethylene insert were utilized to obtain in vivo, six-degrees-of-freedom positions and orientation of the models using the JointTrack program (sourceforge.net/projects/jointtrack) (Fig. 3). The optical geometry of the fluoroscopic system (principal distance, principal point) was determined based on images of a calibration target. An implant surface model was projected onto the geometry-corrected fluoroscopic images, and its 3D position and rotation was iteratively adjusted so that its silhouette matched with that of the knee prosthesis using the JointTrack. After the tibial component was registered, the bead model was first fitted onto the upper surface of the tibial tray and then fitted with the beads in the fluoroscopic image by rotating around the Y rotation axis (or tibial vertical axis).

**Fig. 4:**
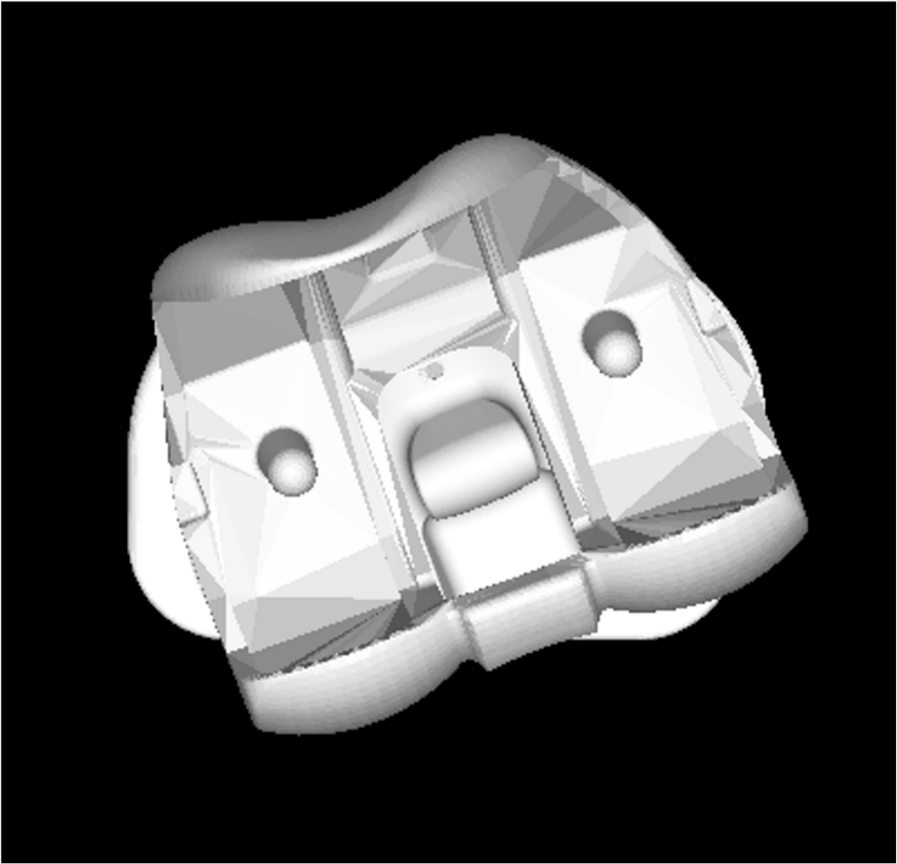
3D-JointManager software (GLAB Corp.) was used to compute 6 degrees-of-freedom positions and orientations of the CAD models at each frame. This figure showes external rotation offset of the mobile-insert relative to the tibial tray and very small rotation of the femoral component relative to the insert

Following the registration process, relative motions of the three CAD models were computed using the 3D-JointManager software (GLAB Inc., Japan) (Fig. 4). Rotation of the femoral component relative to the tibial component (FEM/TIB) or polyethylene insert (FEM/INS), and rotation of the polyethylene insert relative to the tibial component (INS/TIB), were computed using a joint coordinate system proposed by Andriacchi et al. [23]. Rotational offset was measured at maximal extension position at each time point in each subject. External rotation of the superior component relative to the inferior was denoted as positive.

### Internal validity

To analyze knee kinematics during a squatting activity, we selected 20 frames from maximal knee extension to maximal knee flexion. Kinematics was analyzed at 5° increments of knee flexion after B-spline curve approximation was performed. In the knee joint, this matching method has an estimated accuracy of 0.53 mm for in-plane translation, 1.6 mm for out-plane translation, and 0.54° for rotation [22, 24].

### Statistical analysis

Characteristics and kinematic parameters of subjects were summarized using means and 95% confidence intervals [95% CI] for continuous values. The assumption of normality and equality of variance was assessed using the Shapiro-Wilk test. If the data met parametric assumptions, we used repeated measure ANOVA and Tukey post hoc test for pairwise comparisons. Paired *t* test was employed to examine differences in kinematics between 10 weeks and 1 year postoperatively. If the data did not meet parametric assumptions, Wilcoxon signed-rank test was employed. Out of plane translation (medial-lateral) was excluded from the analyses in this study due to the limited accuracy. Pearson’s correlation coefficient was performed for the relationship between weight and rotation motion of each component. All data were analyzed using SPSS Statistics v21 (SPSS Inc., Chicago, IL) with the significance level set at alpha = .05.

## Results

Thirteen knees of 11 subjects (3 males and 8 females) were evaluated in this study (mean [95% CI] age 71.4 [67.5, 75.3] years, height 153.4 [148.8, 158.1] cm, weight 67.4 [58.9, 75.8] kg, body mass index (BMI) 28.4 [26.3, 30.5] kg/m^2^ at 10 weeks postoperatively) (Table 1).

**Table 1:** Demographic data of study participants

	10 weeks postoperatively	1 year postoperatively	*p* value
Age (years)	71.4 [67.5, 75.3]		
Height (cm)	153.4 [148.8, 158.1]		
Weight (kg)	67.4 [58.9, 75.8]		
BMI (kg/m^2^)	28.4 [26.3, 30.5]		
Active-ROM (degrees)	99.8 [92.5, 107.1]	92.7 [83.5, 101.9]	0.173
Passive-ROM (degrees)	120.1 [113.0, 127.2]	123.8 [111.9, 134.9]	0.196
WOMAC			
Pain	41 (0-263)	5 (0-99)	**0.002**
Stiffness	44 (5-137)	0 (0-92)	**0.009**
Physical function	337 (69-696)	167 (0-426)	**0.019**
Total score	468 (108-1096)	228 (2-489)	**0.005**

Mean [95% CI] for Age, Height, Weight, BMI and ROM results

Median (range) for WOMAC results

*95% CI* 95% confidence interval, *BMI* body mass index, *ROM* range of motion, *WOMAC* Western Ontario and McMaster Universities Osteoarthritis Index

In the clinical measurements, active and passive ROM of the knee on the operated side were 99.8° [92.5, 107.1] and 120.1° [113.0, 127.2] at 10 weeks and 90.8° [83.5, 101.9] and 123.4° [111.9, 134.9] at 1 year, respectively. There were no statistically significant differences between data at the two time-points for either active ROM (*p* = 0.173) or passive ROM (*p* = 0.196). Median WOMAC scores for pain, stiffness, physical function, and total score using 100 mm VAS were 41, 44, 337, and 468 at 10 weeks and those at 1 year were 5, 0, 167, and 228, respectively. All scores at 1 year were significantly less than those at 10 weeks (pain, *p* = 0.002; stiffness, *p* = 0.009; physical function, *p* = 0.019; total score, *p* = 0.005) (Table 1).

The average flexion angles of the tibial and femoral components at the start of movement (maximal extension) were − 2.0° [− 5.5, 1.6] at 10 weeks and − 3.2° [− 10.9, 4.4] at 1 year, respectively. FEM/TIB, FEM/INS, and INS/TIB angles at 10 weeks were 6.0° [2.0, 9.9], − 0.3° [− 1.0, 0.4], and 6.3° [2.1, 10.4], respectively. FEM/INS was significantly smaller than INS/TIB (*p* = 0.013). FEM/TIB, FEM/INS, and INS/TIB angles at 1 year were 4.0° [0.8, 7.2], − 0.8° [− 2.4, 0.7], and 4.9° [1.4, 8.3], respectively. FEM/INS was significantly smaller than INS/TIB (*p* = 0.011). FEM/TIB at 1 year was significantly smaller than that at 10 weeks (*p* = 0.041) (Table 2).

**Table 2:** Rotational offset of each component at the start of movement

	10 weeks postoperatively	1 year postoperatively	*p* values
Knee flexion angle (degrees)	− 2.0 [− 5.5, 1.6]	− 3.2 [− 10.9, 4.4]	0.709
FEM/TIB (degrees)	6.0 [2.0, 9.9]	4.0 [0.8,7.2]	**0.041**
FEM/INS (degrees)	− 0.3 [− 1.0, 0.4] ※	− 0.8 [− 2.4, 0.7] †	0.539
INS/TIB (degrees)	6.3 [2.1, 10.4] ※	4.9 [1.4, 8.3] †	0.136
*p*-value (FEM/INS with INS/TIB)	**0.013**	**0.011**	

Mean [95% CI]

※FEM/INS was significantly smaller than that of INS/TIB at 10 weeks postoperatively

†FEM/INS was significantly smaller than that of INS/TIB at 1 year postoperatively

*FEM/TIB* rotation of the femoral component relative to the tibial component, *FEM/INS* rotation of the femoral component relative to the polyethylene insert, *INS/TIB* rotation of the polyethylene insert relative to the tibial component, *p value* probability value, *95% CI* 95% confidence interval

Rotation motion during a squatting activity from 2.0 [− 5.5, 1.6] degrees hyperextension to 55.3 [44.6, 66.0] degrees flexion for FEM/TIB, FEM/INS, and INS/TIB at 10 weeks were 5.7° [4.2, 7.3], 5.9° [4.5, 7.2], and 1.8° [1.4, 2.2], respectively. Total rotation motion of FEM/INS was significantly greater than that of INS/TIB (*p* < 0.001). At 1 year, rotation motion during squatting from − 3.2 [− 10.9, 4.4] to 53.9 [48.9, 58.8] degrees were 6.3° [4.3, 7.8], 5.5° [3.8, 7.2], and 1.6° [1.1, 2.2], respectively. Total rotation motion of FEM/INS was significantly greater than that of INS/TIB (*p* < 0.001). There were no statistically significant differences between data obtained at 10 weeks and those at 1 year (*p* = 0.702, *p* = 0.547, and *p* = 0.517, respectively) (Table 3). There was no significant correlation between subject’s weight and rotation motion.

**Table 3:** Rotation excursions during squatting activity

	10 weeks postoperatively	1 year postoperatively	*p* values
Knee extension angle (degrees)	− 2.0 [− 5.5, 1.6]	− 3.2 [− 10.9, 4.4]	0.709
Knee flexion angle (degrees)	55.3 [44.6, 66.0]	57.1 [46.7, 67.6]	0.760
FEM/TIB (degrees)	5.7 [4.2, 7.3]	6.3 [4.3, 7.8]	0.702
FEM/INS (degrees)	5.9 [4.5, 7.2] ※	5.5 [3.8, 7.2] †	0.547
INS/TIB (degrees)	1.8 [1.4, 2.2] ※	1.6 [1.1, 2.2] †	0.517
*p* value (FEM/INS with INS/TIB)	< **0.001**	< **0.001**	

Mean [95% CI]

※FEM/INS was significantly greater than that of INS/TIB at 10 weeks postoperatively

† FEM/INS was significantly greater than that of INS/TIB at 1 year postoperatively

*FEM/TIB* rotation of the femoral component relative to the tibial component, *FEM/INS* rotation of the femoral component relative to the polyethylene insert, *INS/TIB*: rotation of the polyethylene insert relative to the tibial component, *p value* probability value, *95% CI* 95% confidence interval

## Discussion

This study was aimed to determine clinical outcomes and rotational kinematics of TKA components and mobile-bearing insert during a squatting activity in vivo at 10 weeks and 1 year postoperatively. The results demonstrated that WOMAC scores at 1 year were significantly smaller than those at 10 weeks, whereas there were no significant differences in active or passive ROM between the two time-points. On kinematic analyses, the mobile-bearing insert played a role in offsetting rotation alignment by around 6° at the starting position with a very small rotation of less than 2° motion during squatting. Contrary to the hypothesis, however, the amount of INS/TIB rotation was significantly smaller than that of FEM/INS rotation during a squatting activity.

The WOMAC scores improved significantly between 10 weeks and 1 year. We observed that some subjects had local warmth and swelling at 10 weeks and, therefore, inflammation and pain were considered to be the cause of the lower subjective score at that time point. Bonnefoy-Mazure et al. [25] showed that WOMAC improved significantly between 3 months and 1 year after TKA. They observed that patients still had pain and motion limitation at 3 months. In our study, WOMAC scores at 1 year were lower than those of the previous study [25]. However, the TKA procedures in our study were considered successful and knee kinematic data at 10 weeks were considered to stay on the good path to achieve good function at 1 year. Therefore, our kinematic findings are considered applicable to successful TKA procedures using the mobile-bearing insert in general.

Mobile-bearing TKA is commonly expected to have a self-alignment mechanism compensating for rotational offset between the tibial and femoral components. An in vivo kinematic study has shown that self-alignment of the polyethylene bearing typically occurs on the tibial tray which should hypothetically lessen polyethylene surface stresses, minimize post impingement, and increase the potential for enhanced polyethylene longevity [1]. This self-alignment mechanism of the polyethylene bearing on the tibial tray is considered superior to a fixed type in maintaining femoral component-to-insert conformity. However, Okamoto et al. [26] indicated that, although mobile-bearing TKA may have certain advantages over fixed-bearing TKA, for example correction of rotational offset while standing, the kinematics of mobile- and fixed-bearing TKA were not significantly different. Yamazaki et al. [16] demonstrated that the femoral component and the mobile-bearing insert were already rotated externally to a similar degree with respect to the tibial component. In this study, we observed a 6° offset in axial rotation at maximal extension, and the advantage of self-alignment with respect to the mobile-bearing TKA was confirmed. This finding is similar to those of previous studies, and rotation offset of the mobile-bearing insert can effectively serve to improve the conformity between the femoral component and mobile bearing insert during squatting, which may improve conformity during gait. Therefore, the mobile-bearing insert may reduce contact pressure and delay the wear of the insert over the long term.

The rotation motion of INS/TIB during a squatting activity in this study was significantly smaller than the rotation of FEM/INS. This finding was different from previous studies using other implants that demonstrated greater INS/TIB rotation motion than FEM/INS [14, 16, 17]. Some studies examining the kinematics of the same TKA implant as that used in this study demonstrated rotation of the femoral component on the insert [19, 27, 28]. Moreover, Garling et al. [19] showed variations of rotational kinematics of the insert on the tibial tray including greater rotation and no rotation and indicated that the conformity of this prosthesis was low enough that the femoral component is allowed to translate with respect to the insert without forcing the insert to rotate. In our study, the femoral rotation motion on the insert was clearly greater than the insert rotation motion on tibial tray. Garling et al. [19] measured using step-up motion, and we used a squat motion. The result of this study was similar to that of Garling et al. [19]. Both activities were under loading condition, and vertical axial force was applied to the mobile-insert, so that the insert was compressed toward the tibial tray. Since this force acted to increase friction at the contact surface, it might have prevented INS/TIB rotation. Therefore, it remains unclear whether this phenomenon also occurs during a non-loading activity such as leg extension in a seated position. Since the conflicting kinematic findings across studies might be related to variations in the implant design and/or loading conditions, the results have a limited generalizability among implants from various manufacturers.

There were several strengths and limitations in this study. Strengths include (1) the use of a 3D-to-2D registration technique with fluoroscopy, which is an accurate and well-established technique to measure in vivo knee kinematics [22, 24]. Our single plane fluoroscopic method utilized a 17 × 17-in. field of view to allow analysis of the knee during a squatting activity; (2) all analyses were performed by a single investigator to avoid potential inter-investigator errors. Thus, systematic errors between investigators did not influence the results; and (3) because X-rays penetrate the polyethylene insert, we injected metal beads into the polyethylene insert to make it visible. Furthermore, CT images were taken in each subject to confirm the exact locations of the beads. The method using single plane fluoroscopic images is less accurate for out-of-plane kinematics because out-of-plane 3D-pose of models on the screen involved a greater error [24]. Therefore, kinematic analyses of squatting or stair ascending/descending activity in the literature avoided reporting medial/lateral tibial translation. The 3D-to-2D registration technique using dual plane fluoroscopic images is more accurate than analyses using single plane fluoroscopic images but has a smaller radiographic area, which limits the ability to capture greater and more dynamic movement of the joints. Therefore, there is an advantage in utilizing a single plane technique. The limitations of our study include the following: (1) although the sample size was small, this cohort is similar to that of the previous fluoroscopic studies, comparison between FEM/INS and INS/TIB showed a post hoc power of 0.9 or more at 10 week and 1 year after surgery. Thus, we thought to provide reliable data with sufficient sample size for analyses; (2) the 3D-to-2D registration method using single-plane fluoroscopic images provided limited measurement accuracy for out-of-plane kinematics. Therefore, we did not include medial-lateral translation in this study. As for external validity, the results of this study can be generalized to subjects aged 50 years and older who underwent TKA with the same mobile-bearing insert model, but the current data are limited to a Japanese population.

## Conclusions

The mobile-bearing insert improves the rotational offset of the femoral and tibial components at the starting position, but the amount of rotation motion during squatting at the INS/TIB interface is very small. This study is thought to provide useful data for improving surgical procedures, developing future implant designs and understanding the implant wear pattern in TKA with a mobile-bearing. In a future study, it will be necessary to analyze whether the amount of mobile-bearing insert movement is affected by the presence or absence of weight-loading.

## Data Availability

Not applicable.

## Abbreviations

95% CI: 95% confidence interval
BMI: Body mass index
CAD: Computer-added design
FEM/INS: Rotation of the femoral component relative to the polyethylene insert
FEM/TIB: Rotation of the femoral component relative to the tibial component
INS/TIB: Rotation of the polyethylene insert relative to the tibial component
KOA: Knee osteoarthritis
ROM: Range of motion
TKA: Total knee arthroplasty
WOMAC: Western Ontario and McMaster Universities Osteoarthritis Index

## References

[1] Dennis DA, Komistek RD (2006). Mobile-bearing total knee arthroplasty: design factors in minimizing wear. Clin Orthop Relat Res.

[2] Amaro JT, Arliani GG, Astur DC, Debieux P, Kaleka CC, Cohen M (2016). No difference between fixed- and mobile-bearing total knee arthroplasty in activities of daily living and pain: a randomized clinical trial. Knee Surg Sports Traumatol Arthrosc.

[3] Bistolfi A, Massazza G, Lee GC, Deledda D, Berchialla P, Crova M (2013). Comparison of fixed and mobile-bearing total knee arthroplasty at a mean follow-up of 116 months. J Bone Joint Surg Am.

[4] Bo ZD, Liao L, Zhao JM, Wei QJ, Ding XF, Yang B (2014). Mobile bearing or fixed bearing? A meta-analysis of outcomes comparing mobile bearing and fixed bearing bilateral total knee replacements. Knee.

[5] Fransen BL, van Duijvenbode DC, Hoozemans MJM, Burger BJ (2016). No differences between fixed- and mobile-bearing total knee arthroplasty. Knee Surg Sports Traumatol Arthrosc.

[6] Hofstede SN, Nouta KA, Jacobs W, van Hooff ML, Wymenga AB, Pijls BG, et al. Mobile bearing vs fixed bearing prostheses for posterior cruciate retaining total knee arthroplasty for postoperative functional status in patients with osteoarthritis and rheumatoid arthritis. Cochrane Database Syst Rev. 2015;(2):Cd003130. 10.1002/14651858.CD003130.pub3 PMC10960232 25650566

[7] Li YL, Wu Q, Ning GZ, Feng SQ, Wu QL, Li Y (2014). No difference in clinical outcome between fixed- and mobile-bearing TKA: a meta-analysis. Knee Surg Sports Traumatol Arthrosc.

[8] Mahoney OM, Kinsey TL, D'Errico TJ, Shen J (2012). The John Insall award: no functional advantage of a mobile bearing posterior stabilized TKA. Clin Orthop Relat Res.

[9] Marques CJ, Daniel S, Sufi-Siavach A, Lampe F (2015). No differences in clinical outcomes between fixed- and mobile-bearing computer-assisted total knee arthroplasties and no correlations between navigation data and clinical scores. Knee Surg Sports Traumatol Arthrosc.

[10] Poirier N, Graf P, Dubrana F (2015). Mobile-bearing versus fixed-bearing total knee implants. Results of a series of 100 randomised cases after 9 years follow-up. Orthop Traumatol Surg Res.

[11] Schotanus MGM, Pilot P, Kaptein BL, Draijer WF, Tilman PBJ, Vos R (2016). No difference in terms of radiostereometric analysis between fixed- and mobile-bearing total knee arthroplasty: a randomized, single-blind, controlled trial. Knee Surg Sports Traumatol Arthrosc.

[12] Shi X, Shen B, Yang J, Kang P, Zhou Z, Pei F (2014). In vivo kinematics comparison of fixed- and mobile-bearing total knee arthroplasty during deep knee bending motion. Knee Surg Sports Traumatol Arthrosc.

[13] Watanabe T, Ishizuki M, Muneta T, Banks SA (2012). Matched comparison of kinematics in knees with mild and severe varus deformity using fixed- and mobile-bearing total knee arthroplasty. Clin biomech (Bristol, Avon).

[14] Futai K, Tomita T, Yamazaki T, Tamaki M, Yoshikawa H, Sugamoto K (2011). In vivo kinematics of mobile-bearing total knee arthroplasty during deep knee bending under weight-bearing conditions. Knee Surg Sports Traumatol Arthrosc.

[15] Kurita M, Tomita T, Yamazaki T, Fujii M, Futai K, Shimizu N (2012). In vivo kinematics of high-flex mobile-bearing total knee arthroplasty, with a new post-cam design, in deep knee bending motion. Int Orthop.

[16] Yamazaki T, Futai K, Tomita T, Sato Y, Yoshikawa H, Tamura S (2015). 3D kinematics of mobile-bearing total knee arthroplasty using X-ray fluoroscopy. Int J Comput Assist Radiol Surg.

[17] Zingde SM, Leszko F, Sharma A, Mahfouz MR, Komistek RD, Dennis DA (2014). In vivo determination of cam-post engagement in fixed and mobile-bearing TKA. Clin Orthop Relat Res.

[18] Dennis DA, Komistek RD, Mahfouz MR, Outten JT, Sharma A (2005). Mobile-bearing total knee arthroplasty: do the polyethylene bearings rotate?. Clin Orthop Relat Res.

[19] Garling EH, Kaptein BL, Nelissen RG, Valstar ER (2007). Limited rotation of the mobile-bearing in a rotating platform total knee prosthesis. J Biomech.

[20] Bellamy N, Buchanan WW, Goldsmith CH, Campbell J, Stitt LW (1988). Validation study of WOMAC: a health status instrument for measuring clinically important patient relevant outcomes to antirheumatic drug therapy in patients with osteoarthritis of the hip or knee. J Rheumatol.

[21] Miyaji T, Gamada K, Kidera K, Ikuta F, Yoneta K, Shindo H (2012). In vivo kinematics of the anterior cruciate ligament deficient knee during wide-based squat using a 2D/3D registration technique. J Sports Sci Med.

[22] Banks SA, Hodge WA (1996). Accurate measurement of three-dimensional knee replacement kinematics using single-plane fluoroscopy. IEEE Trans Biomed Eng.

[23] Andriacchi TP, Johnson TS, Hurwitz DE, Matarajan RN, Mow VC, Huiskes R (2005). Musculoskeletal dynamics, locomotion, and clinical applications. Basic orthopaedic biomechanics & mechano-biology, third edition.

[24] Moro-oka TA, Hamai S, Miura H, Shimoto T, Higaki H, Fregly BJ (2007). Can magnetic resonance imaging-derived bone models be used for accurate motion measurement with single-plane three-dimensional shape registration?. J Orthop Res.

[25] Bonnefoy-Mazure A, Armand S, Sagawa Y, Suva D, Miozzari H, Turcot K (2017). Knee kinematic and clinical outcomes evolution before, 3 months, and 1 year after total knee arthroplasty. J Arthroplast.

[26] Okamoto N, Nakamura E, Nishioka H, Karasugi T, Okada T, Mizuta H (2014). In vivo kinematic comparison between mobile-bearing and fixed-bearing total knee arthroplasty during step-up activity. J Arthroplast.

[27] Garling EH, Wolterbeek N, Velzeboer S, Nelissen RGHH, Valstar ER, Doorenbosch CAM (2008). Co-contraction in RA patients with a mobile bearing total knee prosthesis during a step-up task. Knee Surg Sports Traumatol Arthrosc.

[28] Wolterbeek N, Garling EH, Mertens B, Valstar ER, Nelissen RG (2009). Mobile bearing knee kinematics change over time. A fluoroscopic study in rheumatoid arthritis patients. Clin Biomech.

